# M2 macrophage is the predominant phenotype in airways inflammatory lesions in patients with granulomatosis with polyangiitis

**DOI:** 10.1186/s13075-017-1310-4

**Published:** 2017-05-18

**Authors:** Alexandre Wagner Silva de Souza, Mirjan van Timmeren, Jan-Stephan Sanders, Coen Stegeman, Peter Heeringa, Cees G. M. Kallenberg, Johanna Westra

**Affiliations:** 1 Department of Rheumatology and Clinical Immunology, University Medical Center Groningen, University of Groningen, Groningen, The Netherlands; 2 Rheumatology Division, Universidade Federal de São Paulo - Escola Paulista de Medicina, São Paulo, SP Brazil; 3 Department of Pathology and Medical Biology, University Medical Center Groningen, University of Groningen, Groningen, The Netherlands; 4 Nephrology Division, Department of Internal Medicine, University Medical Center Groningen, University of Groningen, Groningen, The Netherlands

**Keywords:** Granulomatosis with polyangiitis, Macrophages, granuloma, Antineutrophil cytoplasmic antibodies, T cells

## Abstract

**Background:**

Macrophages may present two distinct phenotypes indicated as M1 and M2 under different stimuli. M1 and M2 macrophages have divergent functions that range from enhancement of inflammation for M1 to tissue repair and remodeling for M2 macrophages. The objective of this study was to evaluate the distribution of M1 and M2 macrophage phenotypes in biopsies from the airways of patients with active granulomatosis with polyangiitis (GPA) and to analyze their associations with T and B cells in those biopsies, and with nasal carriage of *Staphylococcus aureus,* disease parameters and therapy.

**Methods:**

Consecutive GPA patients (n = 35) with active airway disease, who underwent respiratory tract biopsy were included. Immunohistochemical evaluation was performed to assess the distribution of macrophages and T and B cells using the markers CD68, CD3 and CD20, respectively. CD86 was used as the M1 marker and CD163 as the M2 marker while Tbet and GATA-3 were used as Th1 and Th2 markers, respectively. At the time of the biopsy patients were assessed for nasal carriage of *Staphylococcus aureus* and treatment.

**Results:**

Percentages of macrophages and T cells were significantly higher than those of B cells in lesional tissue from the respiratory tract in GPA. M2 macrophages and Th2 cells were more frequent than M1 macrophages (*p* = 0.0007) and Th1 cells (*p* < 0.0001), respectively. Percentages of T cells were higher in nose biopsies than in biopsies from other sites (*p* = 0.021); macrophages and CD163^+^ macrophages were more predominant in biopsy sites other than the nose (*p* = 0.039 and *p* = 0.012, respectively). Carriage of *Staphylococcus aureus* was associated with higher T cell scores (*p* = 0.014). The frequency of macrophages, especially M2 macrophages, was higher in GPA patients treated with immunosuppressive agents (*p* = 0.010); daily prednisolone dose was positively correlated with all macrophage markers. However, in multivariate analysis no independent associations were found between disease parameters and therapy with macrophage markers or T cells.

**Conclusion:**

In GPA, M2 is the predominant macrophage phenotype in the respiratory tract. Although some associations were observed between macrophages and T cells with therapy and nasal carriage of *Staphylococcus aureus*, they were not independently significant in multivariate analysis.

**Electronic supplementary material:**

The online version of this article (doi:10.1186/s13075-017-1310-4) contains supplementary material, which is available to authorized users.

## Background

Granulomatosis with polyangiitis (GPA) is a type of antineutrophil cytoplasmic antibody (ANCA)-associated vasculitis characterized by necrotizing granulomatous inflammation and small to medium-sized vessel vasculitis, which may involve multiple organs [1, 2]. In GPA, granulomatous inflammation usually involves the upper and lower respiratory tract and the orbits and meninges, leading to mass formation (e.g. pulmonary nodules or masses, orbital masses, subglottic and/or bronchial stenosis, hypertrophic pachimeningitis) and destructive lesions such as bony erosions of the sinuses, saddle nose, septal perforation or cavitation of pulmonary nodules/masses [3, 4]. Once regarded as a relatively harmless phenotype of GPA due to its expression mainly in patients with localized disease, recent studies have shown that necrotizing granulomatous inflammation may lead to significant morbidity and even death in GPA, and it is often refractory to conventional treatment [5, 6].

Macrophages are cells of the innate immune system that play an important role in inflammation, host defense, tissue remodeling and regulation of metabolism [7]. The plasticity of macrophages is highlighted by their capacity to change their functional phenotypes in response to distinct stimuli. Stimulation by toll-like receptor (TLR) ligands or by interferon (IFN)-γ triggers M1 polarization, while the exposure to cytokines like interleukin (IL)-4 and/or IL-13 leads to M2 polarization, mirroring T helper (Th) Th1/Th2 polarization of T cells [8]. M1 macrophages usually express IL-12 and IL-23, produce effector molecules (e.g. reactive oxygen and nitrogen species) and inflammatory cytokines such as IL-1β, tumor necrosis factor α (TNFα) and IL-6. These M1 macrophages initiate an inflammatory response and mediate tissue damage, and they are responsible for resistance to intracellular pathogens and tumor cells. In contrast, M2 macrophages express IL-10 and a high level of scavenger-like, mannose-like and galactose-like receptors. M2 macrophages are involved in inhibiting inflammation, in tissue repair and remodeling and in angiogenesis and tumor progression [7, 8]. Nonetheless, M2 macrophages are further sub classified into four different additional phenotypes as follows: M2a, M2b, M2c and M2d, with distinct functions. M2a is a wound-healing macrophage that promotes tissue repair and remodeling while M2c is a suppressor macrophage that is responsible for downregulating immune response. M2b macrophage is a pro-inflammatory M2 macrophage that is induced by exposure to immune complexes, TLR ligands or IL-1 receptor antagonist. M2d macrophages produce high levels of IL-10 and vascular endothelial growth factor (VEGF), inhibiting immune response and promoting angiogenesis [9].

Macrophages are key cells in granulomatous inflammation and may be present as epithelioid cells, multinucleated giant cells and in some instances as foam cells [10]. Granulomatous inflammation in GPA is characterized by poorly formed granulomas usually associated with geographic necrosis, micro abscesses and scattered giant cells [11, 12].

In the early stages of ANCA-associated pauci-immune necrotizing glomerulonephritis, CD163^+^ M2 macrophages predominate at sites of fibrinoid necrosis, where they exceed the numbers of neutrophils and T cells. CD68^+^ cells and CD163^+^ macrophages are more frequently found in normal-appearing glomeruli in this condition than in glomeruli of controls with thin basement membrane nephropathy [13]. However, to the best of our knowledge, it is not known whether macrophages found in granulomatous lesions in the upper airways of patients with GPA display an M1 or M2 phenotype. Assessment of their distribution would help to uncover whether granulomatous inflammation in the airways of patients with GPA displays a pro-inflammatory phenotype of macrophages in line with a Th1 response or a more reparative and regulatory phenotype mirroring a predominant Th2 response. We hypothesize that macrophages found in granulomatous lesions in the airways of patients with GPA are predominantly of the M1 phenotype, due to the highly inflammatory nature of these lesions and the Th1-polarized T cell response involved in airway inflammation in patients with GPA. This study aimed to investigate the distribution of macrophage phenotypes found in active granulomatous lesions in the upper airways of patients with GPA and to analyze associations with infiltrating T and B cells from these inflammatory sites and with chronic carriage of *Staphylococcus aureus*, disease parameters and therapy in GPA.

## Methods

### Patients

Thirty-five consecutive patients who were classified as having GPA according to the 1990 American College of Rheumatology (ACR) criteria for GPA (Wegener’s granulomatosis) or according to the European Medicines Agency (EMEA) classification algorithm [14, 15], and underwent a biopsy from the respiratory tract were selected for this study. Disease extension was ascertained according to European Vasculitis Study (EUVAS) disease categorization of ANCA-associated vasculitis [16]. Table 1 describes disease features, biopsy sites, and therapy in those patients with GPA. All biopsies were performed for diagnostic purposes by nasofibroscopy, bronchoscopic procedures or thoracotomy. The majority of patients with GPA underwent nose biopsies; other biopsy sites (one patient per site) included ethmoidal sinus, Eustachian tube, nasopharynx, subglottic area, trachea, bronchus and lung.

**Table 1:** Disease features and biopsy sites in patients with granulomatosis with polyangiitis

Variables	Results (n = 35)
Age, years	48.9 ± 12.6
Female, *n* (%)	22 (62.9)
*Disease status*	
Onset, *n* (%)	9 (25.7)
Relapse, *n* (%)	25 (71.4)
Persistent disease, *n* (%)	1 (2.8)
Years since diagnosis^a^	8.7 ± 5.6
*Disease extension*	
Localized disease, *n* (%)	29 (82.9)
Generalized disease, *n* (%)	6 (17.1)
*Disease manifestations*	
ENT involvement, *n* (%)	34 (97.1)
Eye disease, *n* (%)	8 (22.8)
Lung involvement, *n* (%)	7 (20.0)
Glomerulonephritis, *n* (%)	4 (11.4)
Cutaneous vasculitis, *n* (%)	1 (2.8)
*ANCA positivity*	
cANCA by IIF, *n* (%)	33 (94.3)
PR3-ANCA by ELISA, *n* (%)	32 (91.4)
*Biopsy sites*	
Nose, *n* (%)	28 (80.0)
Other sites, *n* (%)	7 (20.0)
*Staphylococcus aureus*	
Positive culture, *n* (%)^b^	19 (55.9)
*Therapy*	
No therapy, *n* (%)	13 (37.1)
Prednisolone, *n* (%)	10 (28.6)
Prednisolone daily dose, mg	11.2 (9.3–20.0)
Daily co-trimoxazole, *n* (%)	14 (40.0)
Cyclophosphamide, *n* (%)	4 (11.4)
Azathioprine, *n* (%)	4 (11.4)
Methotrexate, *n* (%)	1 (2.8)
Mycophenolate mofetil, *n* (%)	4 (11.4)
Cyclosporin A, *n* (%)	1 (2.8)

Continuous variables are presented as mean and standard deviation or as median and interquartile range as appropriate. *ANCA* antineutrophil cytoplasmic antibodies, *ELISA* enzyme-linked immunosorbent assay, *ENT* ear, nose and throat, *IIF* indirect immunofluorescence, *n* number of patients, *PR3* proteinase 3. ^a^Years since diagnosis includes patients with relapsing and persistent disease. ^b^Results of nasal swab cultures were available for 34 patients

The ANCA test was performed by in-house indirect immunofluorescence (IIF) on ethanol-fixed neutrophil slides and results were displayed as the pattern (i.e. cytoplasmic, perinuclear or atypical ANCA) and ANCA titers. ANCA specificity for anti-proteinase 3 (anti-PR3) and anti-myeloperoxidase (anti-MPO) antibodies was confirmed by an in-house capture enzyme-linked immunosorbent assay (ELISA) as described earlier [17].

### Immunohistochemical evaluation

Biopsy specimens were processed for immunohistochemical evaluation. Paraffin-embedded specimens and formalin-fixed 4-μm sections were deparaffinized and were incubated with 10 mM Tris/HCl and 1 mM EDTA (Ethylenediaminetetraacetic acid) buffer (pH 9.0) at 90 °C for 60 minutes for antigen retrieval. Endogenous peroxidase was blocked with 0.3% H_2_O_2_ in phosphate-buffered saline (PBS) for 30 minutes. Slides were pre-incubated with 20% bovine serum albumin (BSA) and incubated with the following primary antibodies diluted in PBS with 1% BSA: anti-CD3 (Abcam, Bristol, UK), anti-CD20 (Abcam, Bristol, UK), anti-CD68 (Abcam, Bristol, UK), anti-CD86 (Abcam, Bristol, UK) and anti-CD163 (Abcam, Bristol, UK) for 1 hour at room temperature. Binding was detected by appropriate horseradish-peroxidase-labeled secondary antibodies and peroxidase activity was developed using 3, 3’-diaminobenzidine tetrachloride (10 minutes at room temperature). To obtain nuclear staining for the transcription factors Tbet and GATA-3 for Th1 and Th2 cells, respectively, 0.5% Triton was added to wash and incubation buffer and anti-T-bet/Tbx21 clone 4B10 (Abcam, Bristol, UK) and anti-GATA-3 (Abcam, Bristol, UK) antibodies were used. For GATA-3 staining, antigen retrieval was performed with citrate buffer (10 mM, pH 6.0).

All sections were stored digitally after examination using a Nanozoomer Digital Pathology Scanner (NDP Scan U10074-01; Hamamatsu Photonics K.K.) and quantified using the positive pixel count algorithm with ImageScope Viewer software (V11.2.0.780 Aperio; e-Pathology Solution). The percentage of strong positive brown and positive pixels was determined in comparison to the total number of pixels (positive and negative).

### Statistical analysis

Data were analyzed using IBM SPSS Statistics for Windows, version 20.0 (Armonk, USA) and graphs were built using GraphPad Prism version 5.0 for Windows (La Jolla, USA). Categorical variables were presented as total number and percentage while continuous data were presented as mean ± standard deviation or as median and interquartile range as appropriate. Student’s *t* test or the Mann-Whitney *U* test was used for comparison of continuous data between two groups, while comparisons between three or more groups were performed using the Kruskal-Wallis test. Correlation between variables was analyzed using Spearman’s correlation coefficient. Univariate and multivariate models of linear regression were built to analyze associations between clinical and therapy variables with frequencies of CD3, CD20, CD68, CD86 and CD163 in airway biopsies from patients with GPA. Results are expressed as β coefficient, *p* value and *R* ^2^ as appropriate. Statistical significance was set at 5% (*p* < 0.05).

## Results

### Macrophages and lymphocytes in airway lesions in GPA

**Fig. 1:**
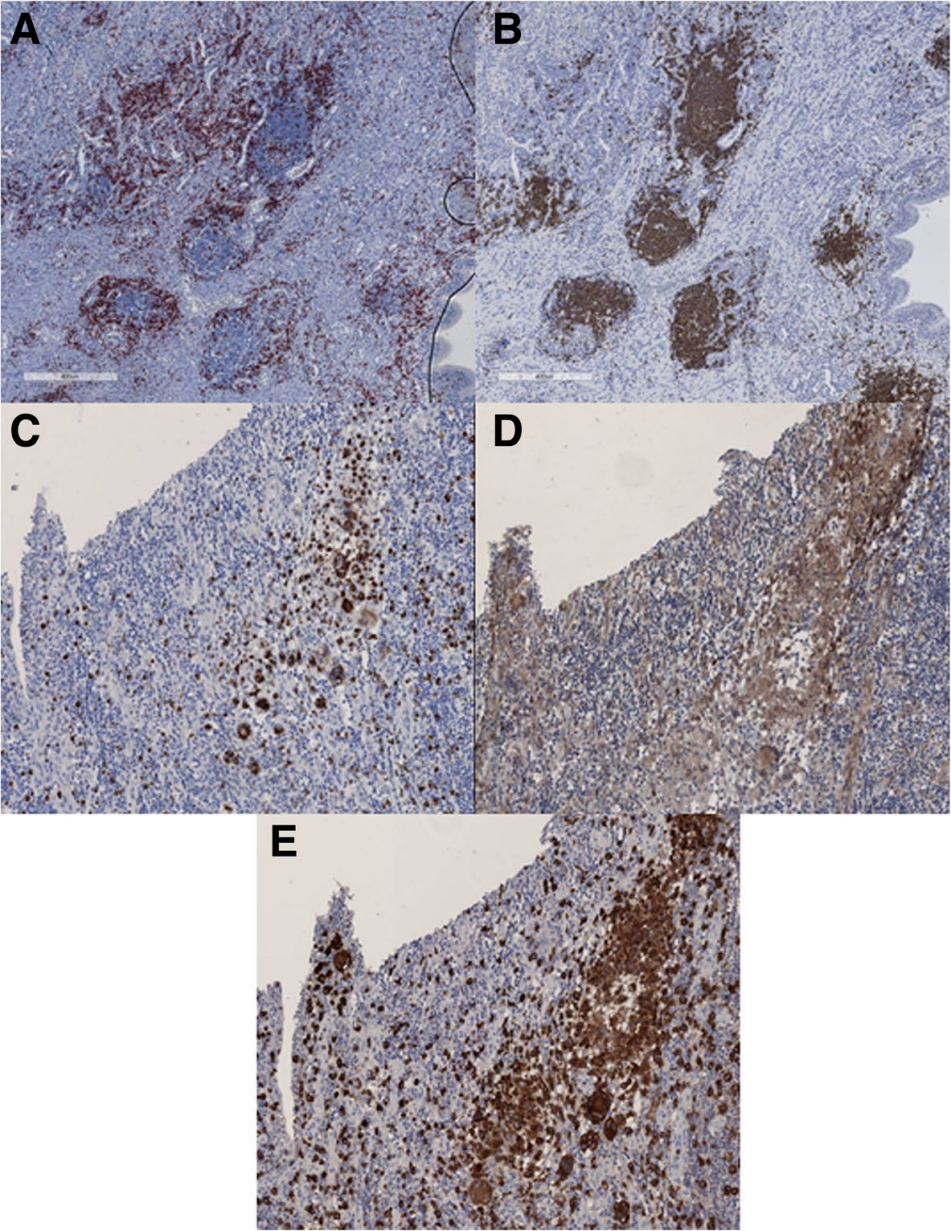
Representative figures of immunostainings of B cells (**a**), T cells (**b**), pan-macrophage marker CD68 (**c**), M1 macrophages (**d**) and M2 macrophages (**e**). B cells and T cells cluster in ectopic lymphoid-like structures with B cells centered and T cells in the periphery. Macrophages are found scattered throughout the inflammatory infiltrate of nasal mucosa of a patient with granulomatosis with polyangiitis

**Fig. 2:**
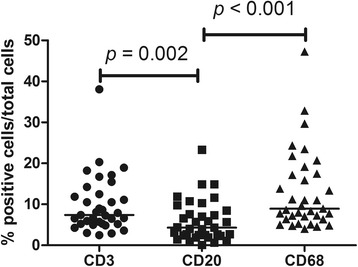
Lymphocytes and macrophages in airway biopsies from patients with granulomatosis with polyangiitis (GPA). The frequency of macrophages and T cells is significantly higher than that of B cells in airway biopsies from patients with GPA. Percentages of T cells and macrophages did not differ significantly

**Fig. 3:**
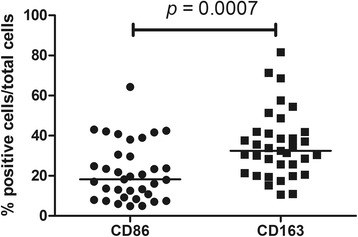
Comparison of M1 and M2 macrophages scores in airway biopsies from patients with granulomatosis with polyangiitis (GPA). M2 macrophages are the predominant macrophage phenotype found in airway biopsies from patients with GPA

**Fig. 4:**
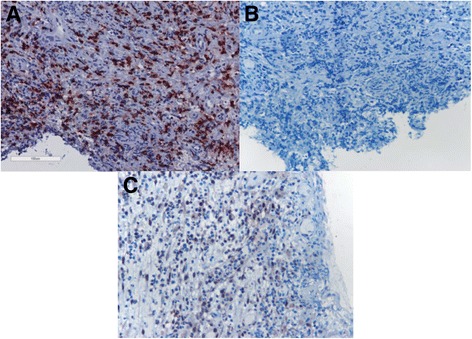
Representative figures of immunostaining of T cells (**a**), T helper (Th)1 (**b**) and Th2 (**c**) cells. The expression of the nuclear transcription factor GATA-3 is (e.g. a Th2 marker) is significantly higher than that of Tbet (e.g. a Th1 marker) in airway biopsies from patients with granulomatosis with polyangiitis

Macrophage markers were more scattered throughout inflammatory infiltrates while B and T cells were gathered in nodules that resembled ectopic lymphoid-like structures (Fig. 1a-e). The percentage of macrophages and T cells was significantly higher than that of B cells in inflammatory lesions of the airways from patients with GPA. No difference was found between percentages of macrophages and T cells (Fig. 2). Amongst macrophages, the frequency of cells expressing the M2 marker CD163 was significantly higher than that of cells expressing the M1 marker CD86 (*p* = 0.0007) (Fig. 3). Similar to what was found for M2 and M1 markers, the expression of the Th2 marker GATA-3 amongst T cells was significantly higher than the expression of the Th1 marker Tbet (1.229 ± 0.598 vs. 0.171 ± 0.382; *p* < 0.0001) (Fig. 4a-c). The frequency of T cells was higher in nose biopsies in comparison with other biopsy sites (*p* = 0.021) whereas the frequency of macrophages and CD163^+^ macrophages was significantly higher in biopsy sites other than the nose (*p* = 0.039 and *p* = 0.012, respectively) (Table 2).

**Table 2:** Distribution of lymphocytes and macrophages in biopsies from different sites of the airways of patients with granulomatosis with polyangiitis

Variables	Nose biopsy (n = 28)	Biopsy from other sites (n = 7)	*p*
CD3, %	8.3 (6.0–15.1)	5.2 (3.0–6.5)	0.021^a^
CD20, %	4.2 (2.4–7.5)	4.3 (1.9–10.7)	0.741
CD68, %	8.4 (6.3–13.6)	20.7 (7.2–29.6)	0.039^a^
CD86, %	16.9 (8.5–24.4)	29.5 (17.0–43.0)	0.114
CD163, %	31.7 ± 13.5	49.0 ± 21.7	0.012^a^

Data are presented as median and interquartile range or as mean and standard deviation. ^a^Significant differences

### Lymphocytes, macrophages, disease parameters and therapy in GPA

**Fig. 5:**
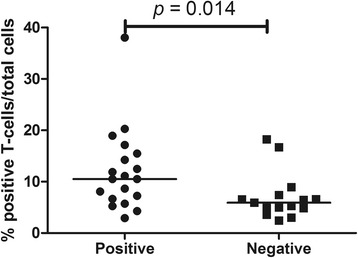
Positive nose culture for *Staphylococcus aureus* and CD3^+^ cells in airway biopsies from patients with granulomatosis with polyangiitis (GPA). Patients with GPA with positive nose cultures for *Staphylococcus aureus* presented with higher scores of T cells in airway biopsies

Airway biopsy scores for T cells, B cells and macrophages were analyzed in relation to disease extension in GPA, disease onset or relapse, biopsy sites and positive nose culture for *Staphylococcus aureus*. No significant differences were found in lymphocytes or macrophages in relation to disease extension or disease onset/relapse (Additional file 1: Table S1). However, a positive nose culture for *Staphylococcus aureus* was associated with significantly higher T cell percentages compared with biopsies from patients with a negative nose culture (10.5% (6.6–15.4) vs. 5.9% (4.8–7.4); *p* = 0.014) (Additional file 1: Table S1 and Fig. 5).

**Fig. 6:**
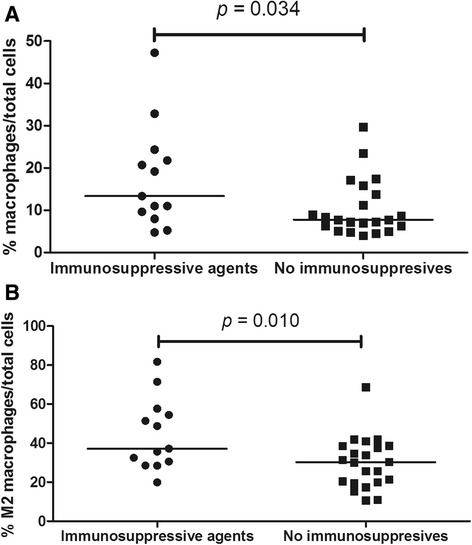
Relationship between immunosuppressive therapy and macrophages (CD68^+^ and CD163^+^ macrophages) in airway biopsies from patients with granulomatosis with polyangiitis (GPA). Higher frequency of macrophages (**a**), especially M2 macrophages (**b**), is observed in airway biopsies from patients with GPA on immunosuppressive therapy compared to patients not on immunosuppressive therapy

There were no differences in T cell, B cell or macrophage scores between patients receiving and not receiving any therapy (i.e. prednisolone and/or immunosuppressive agents) (Additional file 2: Table S2), or between patients receiving or not receiving co-trimoxazole, regardless of whether they were chronic carriers of *Staphylococcus aureus* (Additional file 2: Table S2). We observed a trend towards lower percentages of T cells and higher percentages of M2 macrophages in patients receiving prednisolone compared with those not receiving prednisolone (Additional file 2: Table S2). The percentage of macrophages, especially M2 macrophages, was significantly higher in patients with GPA treated with immunosuppressive agents in comparison to those not treated with these agents (Table 3 and Fig. 6a and b).

**Table 3:** Evaluation of lymphocyte and macrophage scores in relation to the use of immunosuppressive agents in granulomatosis with polyangiitis

Variables	Immunosuppressive agents (n = 13)	No immunosuppressive agents (n = 22)	*p*
CD3, %	8.0 (4.9–16.1)	7.3 (5.7–11.3)	0.891
CD20, %	4.0 (1.2–8.8)	5.0 (2.6–8.7)	0.322
CD68, %	13.3 (8.8–23.0)	7.7 (6.0–14.3)	0.034^a^
CD86, %	29.5 (11.9–41.8)	16.5 (8.2–23.4)	0.082
CD163, %	44.4 ± 18.3	29.8 ± 13.2	0.010^a^

Data are presented as median and interquartile range or as mean and standard deviation. ^a^Significant differences

Distribution of T and B cells and macrophage subsets in airway biopsies from patients with GPA was related to the daily dose of prednisolone. There was significant positive correlation between all macrophage markers and the daily dose of prednisolone, including CD68 (rho = 0.858; *p* = 0.001), CD86 (rho = 0.753; *p* = 0.012) and CD163 (rho = 0.759; *p* = 0.011). However, no correlation was observed between CD3 or CD20 scores and the daily dose of prednisolone (Additional file 3: Table S3).

In univariate analysis, positive nasal culture for *Staphylococcus aureus* was significantly associated with the frequency of T cells in airway biopsies from patients with GPA (β = 4.99; *p* = 0.039; *R* ^2^ = 0.127). However, there was no association could be fond between the use of co-trimoxazole and the frequency of T cells (β = 0.64; *p* = 0.794). All macrophage markers (e.g. CD68, CD86 and CD163) were associated with daily prednisolone dose and with the use of immunosuppressive agents (Additional file 4: Table S4). Different models of multivariate linear regression were built using cell markers (i.e. CD3, CD20, CD68, CD86 and CD163) as the dependent variable and age, female gender, positive nasal culture for *Staphylococcus aureus*, generalized disease, use of co-trimoxazole, daily dose of prednisolone and use of immunosuppressive agents as independent variables. Nonetheless, no significant independent associations were found with any cell marker in airway biopsies from patients with GPA (Additional file 5: Table S5).

## Discussion

In this study, we found that macrophages and T cells are more frequent than B cells in airway lesions of patients with GPA. M2 macrophages were the predominant phenotype, mainly in airway sites other than the nose, and this increase in M2 macrophages was further associated with the use of immunosuppressive agents. Frequencies of macrophages and macrophage subsets were significantly correlated with the daily dose of prednisolone whereas B and T cells were not. Patients with GPA with a positive nose culture for *Staphylococcus aureus* had higher frequencies of T cells infiltrating the nose mucosa.

As macrophage phenotypes mirror Th1 and Th2 functionality [8], and airway inflammation in GPA may be predominantly from a Th1 response, we originally hypothesized that the M1 phenotype would be more frequently found in active lesions in the airways of patients with GPA. However, this hypothesis was proven to be wrong and the expression of the M2 marker CD163 and the Th2 marker GATA-3 was significantly higher in airway lesions from patients with GPA. This unexpected finding may be due to the ongoing Th2 response of patients with GPA evaluated in this study, reflected by the frequent lymphoid follicles observed in parallel with granulomatous airway inflammation and by the high frequency (94.3%) of a positive ANCA test, indicating a shift towards a Th2 response [18]. Indeed, it is worth mentioning that sera from patients with AAV had a tendency to shift towards an M2c phenotype in macrophages regardless of disease activity, whereas sera from health controls led to an M0 phenotype [19]. Alternatively, another mechanism to explain the predominant M2 phenotype in GPA is the influence of therapy on our results; M2c macrophages may be induced by exposure to glucocorticoids [20]. Although in this study only one third of patients with GPA were under therapy with prednisolone at a relatively low mean daily dose, there was a trend toward higher scores of M2 macrophages and lower scores of T cells in airway biopsies from patients with GPA on prednisolone (Additional file 2: Table S2).

A recent study showed an increase in CD163^+^ M2 macrophages in early lesions of ANCA-associated pauci-immune necrotizing glomerulonephritis, mostly patients with either PR3- or MPO-ANCA. However, it is not possible to associate this finding with therapy, because no information was provided about the use of glucocorticoids or immunosuppressive agents [13]. In addition Park et al. found abundant expression of CD163^+^ cells in granulomatous lesions in the lungs in patients with GPA [21]. Together with our findings, these results indicate that M2 polarization is observed in different tissues affected by GPA, including the upper airways, lungs and kidneys.

Macrophage polarization has been evaluated in tissues from patients with different disorders. Every disease shows a unique pattern of macrophage polarization that may be influenced by the underlying pathophysiologic process [8, 22]. The M1 phenotype is predominantly found in adipose tissue in obese patients, in synovial tissue in patients with rheumatoid arthritis, and in atherosclerotic plaques [9, 22–28]. M2 macrophages are predominantly found in muscle biopsies from patients with neuromuscular sarcoidosis, in lung tissue from patients with idiopathic pulmonary fibrosis and from patients with asthma, and in synovial tissue from patients with osteoarthritis and patients with spondyloarthritis [23, 24, 29–32]. In cancer, M1-polarized macrophages have anti-tumoral activity while infiltration of M2 macrophages in tumoral lesions is associated with tumor progression and with a worse prognosis [32]. Surface markers have been used to identify M1 and M2 macrophages in different studies. The most common markers used for M1 macrophages are CD80 and CD86, whereas CD163 and mannose receptor (also known as CD206) are used to characterize M2 macrophages [9].

In this study, we found higher percentages of CD86 and CD163, specific M1 and M2 markers, respectively, compared with the pan-macrophage marker (CD68). The possible reason for this may be the cytoplasmic distribution of CD68 in contrast with the membrane staining of CD86 and CD163 [33, 34]. Moreover, it is worth mentioning that this was not a cellular count but a pixel count of brown staining from immunohistochemistry slides. In fact, some studies evaluating CD68 and CD163 by immunohistochemical evaluation also found higher scores for CD163 compared with CD68 [12, 35–39].

Macrophages are polarized toward the M1 phenotype in the early phases of bacterial infections, but when infection becomes chronic or upon resolution and convalescence, M2 macrophages prevail in order to prevent excessive tissue damage [21]. Chronic carriage of *Staphylococcus aureus* was included in this study due to its high prevalence in patients with GPA and its impact on the risk of relapse [40]. Moreover, it would influence macrophage polarization in inflammatory lesions of the airways in GPA. Although in chronic rhinosinusitis with nasal polyps an increased number of M2 macrophages and decreased phagocytosis is associated with the persistence of *Staphylococcus aureus* [41], we did not find any association between an increased number of M2 macrophages in nasal mucosa from patients with GPA and nasal carriage of *Staphylococcus aureus*. In the present study, the association found between *Staphylococcus aureus* and T cells in nasal mucosa could indicate that these bacteria trigger local T cell expansion, as staphylococcal super-antigens could be responsible for polyclonal T cell activation and an increased risk of relapse, especially strains of *Staphylococcus aureus* that produce the tsst-1 (toxic shock syndrome toxin-1) super-antigen [40, 42].

Amongst lymphocytes and macrophages, B cells were the cells least present in granulomatous lesions in the airways of patients with GPA. It is known in GPA that granulomas harbor B cells in germinal center-like lymphoid structures, and these cells are considered to be pathogenic ANCA-secreting cells [43]. Activated B cells are found alongside PR3-expressing cells and cells expressing B cell activating factor (BAFF) and a proliferation-inducing ligand (APRIL) in nasal mucosa from patients with GPA [44]. Nonetheless, in this study we did not find any associations between B cells and disease parameters.

Limitations of this study include mainly its cross-sectional nature. Longitudinal evaluation of macrophage phenotypes in airway infiltrates from patients with GPA (i.e. repeated biopsies of nasal mucosa) prior to commencing therapy and after remission is obtained would yield better understanding about the influence of disease activity and therapy. Another limitation is the heterogeneity of biopsy sites evaluated in this study. Even though most biopsies were performed from nasal mucosa, other airway sites could behave in a different way under the influence of different local factors.

## Conclusion

M2 is the predominant macrophage phenotype in the inflammatory infiltrate in the airways of patients with GPA, especially in sites other than the nasal mucosa. In addition, as a mirror of M2 macrophages, the Th2 response is also predominant over the Th1 response in the airways of patients with GPA. Some associations were found between daily prednisolone dose and all macrophage markers, between M2 macrophages and immunosuppressive therapy and between nasal carriage of *Staphylococcus aureus* and an increased number of T cells in the airways of patients with GPA. However, in multivariate analysis they were not shown to be independent from other factors such as age, gender, disease extension or therapy with co-trimoxazole.

## Additional files

Additional file 1: Table S1. Analysis of lymphocytes and macrophages in relation to disease parameters and nasal culture in GPA. Frequencies of CD68, CD86, CD163, CD3 and CD20 were compared between patients with GPA with localized and generalized disease and between patients with GPA who underwent airways biopsy at disease onset and during a disease relapse; no significant differences were observed. Evaluation of lymphocyte and macrophage scores in relation to positive and negative nose cultures for *S. aureus* yielded a significantly higher frequency of T cells in airway lesions from patients with GPA with a positive nasal culture for *S. aureus* compared with patients with GPA with a negative nasal culture for *S. aureus*. No significant differences were observed in B cell or macrophage frequencies in positivity of nasal culture for *S. aureus* in the airways of patients with GPA. (PDF 248 kb)

Additional file 2: Table S2. Evaluation of lymphocyte and macrophage scores in relation to therapy for GPA. Comparisons between patients with GPA who were not on therapy and patients with GPA who were on either prednisolone or immunosuppressive agents did not yield significant results for CD3^+^, CD20^+^, CD68^+^, CD86^+^ or CD163^+^ cells in the airways. Evaluation of lymphocyte and macrophage scores in relation to the use of daily co-trimoxazole in patients with GPA with positive nose cultures for *Staphylococcus aureus*. No significant differences in CD3^+^, CD20^+^, CD68^+^, CD86^+^ or CD163^+^ cells in the airways were found between patients with GPA who were or were not on daily co-trimoxazole. Evaluation of lymphocyte and macrophage scores in relation to the use of daily co-trimoxazole in patients with GPA with negative nose cultures for *S. aureus*; no significant differences were found in CD3^+^, CD20^+^, CD68^+^, CD86^+^ or CD163^+^ cells in the airways between patients with GPA who were or were not on daily co-trimoxazole. Evaluation of lymphocyte and macrophage scores in relation to the use of prednisolone in patients with GPA; no significant differences in CD3^+^, CD20^+^, CD68^+^, CD86^+^ or CD163^+^ cells in the airways were found between patients with GPA who were or were not on prednisolone. (PDF 153 kb)

Additional file 3: Table S3. Correlation between prednisolone daily dose and lymphocytes and macrophages in airway biopsies from patients with GPA. Significant correlation was found between daily dose of prednisolone in patients with GPA and all macrophage markers. (PDF 235 kb)

Additional file 4: Table S4. Univariate linear regression to analyze associations between macrophage markers and therapy in patients with GPA. Significant associations were found between all macrophage markers and daily dose of prednisolone and the use of immunosuppressive agents. (PDF 316 kb)

Additional file 5: Table S4. Multivariate linear regression models to analyze associations between CD3, CD20, CD68, CD86 and CD163 scores in airway biopsies and disease parameters/therapy in patients with GPA. No significant associations were observed between macrophages, B cell or T cell markers with age, gender, positive nasal culture for *S. aureus*, disease extension, use of co-trimoxazole, daily dose of prednisolone or use of immunosuppressive agents. (PDF 240 kb)

## Abbreviations

ACR: American College of Rheumatology
ANCA: Antineutrophil cytoplasmic antibody
anti-MPO: Anti-myeloperoxidase
anti-PR3: Anti-proteinase 3
APRIL: A proliferation-inducing ligand
BAFF: B cell activating factor
BSA: Bovine serum albumin
BVAS: Birmingham Vasculitis Activity Score
CD: Cluster of differentiation
EDTA: Ethylenediaminetetraacetic acid
ELISA: Enzyme-linked immunosorbent assay
EMEA: European Medicines Agency
EUVAS: European Vasculitis Study
GPA: Granulomatosis with polyangiitis
H_2_O_2_: Hydrogen peroxide
IBM: International Business Machine
IFN: Interferon
IIF: Indirect immunofluorescence
IL: Interleukin
PBS: Phosphate-buffered saline
SPSS: Statistical Package for the Social Sciences
Th: T helper
TLR: Toll-like receptor
TNFα: Tumor necrosis factor α
tsst-1: Toxic shock syndrome toxin-1
UK: United Kingdom
USA: United States of America

## References

[1] Jennette JC, Falk RJ, Bacon PA, Basu N, Cid MC, Ferrario F (2013). 2012 revised International Chapel Hill Consensus Conference Nomenclature of Vasculitides. Arthritis Rheum..

[2] Hoffman GS, Kerr GS, Leavitt RY, Hallahan CW, Lebovics RS, Travis WD (1992). Wegener granulomatosis: an analysis of 158 patients. Ann Intern Med..

[3] Boudes P (1990). Purely granulomatous Wegener’s granulomatosis: a new concept for an old disease. Semin Arthritis Rheum..

[4] Polychronopoulos VS, Prakash UB, Golbin JM, Edell ES, Specks U (2007). Airway involvement in Wegener's granulomatosis. Rheum Dis Clin North Am..

[5] Holle JU, Gross WL, Holl-Ulrich K, Ambrosch P, Noelle B, Both M (2010). Prospective long-term follow-up of patients with localised Wegener's granulomatosis: does it occur as persistent disease stage?. Ann Rheum Dis..

[6] Holle JU, Dubrau C, Herlyn K, Heller M, Ambrosch P, Noelle B (2012). Rituximab for refractory granulomatosis with polyangiitis (Wegener's granulomatosis): comparison of efficacy in granulomatous versus vasculitic manifestations. Ann Rheum Dis..

[7] Mantovani A, Biswas SK, Galdiero MR, Sica A, Locati M (2013). Macrophage plasticity and polarization in tissue repair and remodelling. J Pathol..

[8] Sica A, Mantovani A (2012). Macrophage plasticity and polarization: in vivo veritas. J Clin Invest..

[9] Colin S, Chinetti-Gbaguidi G, Staels B (2014). Macrophage phenotypes in atherosclerosis. Immunol Rev..

[10] Ramakrishnan L (2012). Revisiting the role of the granuloma in tuberculosis. Nat Rev Immunol..

[11] Devaney KO, Travis WD, Hoffman G, Leavitt R, Lebovics R, Fauci AS (1990). Interpretation of head and neck biopsies in Wegener's granulomatosis. A pathologic study of 126 biopsies in 70 patients. Am J Surg Pathol.

[12] Jennette JC (2011). Nomenclature and classification of vasculitis: lessons learned from granulomatosis with polyangiitis (Wegener's granulomatosis). Clin Exp Immunol..

[13] Zhao L, David MZ, Hyjek E, Chang A, Meehan SM (2015). M2 macrophage infiltrates in the early stages of ANCA-associated pauci-immune necrotizing GN. Clin J Am Soc Nephrol..

[14] Leavitt RY, Fauci AS, Bloch DA, Michel BA, Hunder GG, Arend WP (1990). The American College of Rheumatology 1990 criteria for the classification of Wegener's granulomatosis. Arthritis Rheum..

[15] Watts R, Lane S, Hanslik T, Hauser T, Hellmich B, Koldingsnes W (2007). Development and validation of a consensus methodology for the classification of the ANCA-associated vasculitides and polyarteritis nodosa for epidemiological studies. Ann Rheum Dis..

[16] Mukhtyar C, Guillevin L, Cid MC, Dasgupta B, de Groot K, Gross W (2009). European Vasculitis Study Group. EULAR recommendations for the management of primary small and medium vessel vasculitis. Ann Rheum Dis.

[17] de Joode AA, Roozendaal C, van der Leij MJ, Bungener LB, Sanders JS, Stegeman CA (2014). Performance of two strategies for urgent ANCA and anti-GBM analysis in vasculitis. Eur J Intern Med..

[18] Abdulahad WH, Lamprecht P, Kallenberg CG (2011). T-helper cells as new players in ANCA-associated vasculitides. Arthritis Res Ther..

[19] Ohlsson SM, Linge CP, Gullstrand B, Lood C, Johansson A, Ohlsson S (2014). Serum from patients with systemic vasculitis induces alternatively activated macrophage M2c polarization. Clin Immunol..

[20] Sica A, Sozzani S, Allavena P, Vecchi A, Locati M (2004). The chemokine system in diverse forms of macrophage activation and polarization. Trends Immunol..

[21] Park J, Lee EB, Song YW (2014). Decreased tumour necrosis factor-α production by monocytes of granulomatosis with polyangiitis. Scand J Rheumatol..

[22] Liu YC, Zou XB, Chai YF, Yao YM (2014). Macrophage polarization in inflammatory diseases. Int J Biol Sci..

[23] Ambarus CA, Noordenbos T, de Hair MJ, Tak PP, Baeten DL (2012). Intimal lining layer macrophages but not synovial sublining macrophages display an IL-10 polarized-like phenotype in chronic synovitis. Arthritis Res Ther..

[24] Tsuneyoshi Y, Tanaka M, Nagai T, Sunahara N, Matsuda T, Sonoda T (2012). Functional folate receptor beta-expressing macrophages in osteoarthritis synovium and their M1/M2 expression profiles. Scand J Rheumatol..

[25] Stöger JL, Gijbels MJ, van der Velden S, Manca M, van der Loos CM, Biessen EA (2012). Distribution of macrophage polarization markers in human atherosclerosis. Atherosclerosis..

[26] Cho KY, Miyoshi H, Kuroda S, Yasuda H, Kamiyama K, Nakagawara J (2013). The phenotype of infiltrating macrophages influences arteriosclerotic plaque vulnerability in the carotid artery. J Stroke Cerebrovasc Dis..

[27] Hill AA, Reid Bolus W, Hasty AH (2014). A decade of progress in adipose tissue macrophage biology. Immunol Rev..

[28] Fjeldborg K, Pedersen SB, Møller HJ, Christiansen T, Bennetzen M, Richelsen B (2014). Human adipose tissue macrophages are enhanced but changed to an anti-inflammatory profile in obesity. J Immunol Res..

[29] Melgert BN, ten Hacken NH, Rutgers B, Timens W, Postma DS, Hylkema MN (2011). More alternative activation of macrophages in lungs of asthmatic patients. J Allergy Clin Immunol..

[30] Pechkovsky DV, Prasse A, Kollert F, Engel KM, Dentler J, Luttmann W (2010). Alternatively activated alveolar macrophages in pulmonary fibrosis-mediator production and intracellular signal transduction. Clin Immunol..

[31] Prokop S, Heppner FL, Goebel HH, Stenzel W (2011). M2 polarized macrophages and giant cells contribute to myofibrosis in neuromuscular sarcoidosis. Am J Pathol..

[32] Chanmee T, Ontong P, Konno K, Itano N (2014). Tumor-associated macrophages as major players in the tumor microenvironment. Cancers (Basel).

[33] de Villiers WJ, Smart EJ (1999). Macrophage scavenger receptors and foam cell formation. J Leukoc Biol..

[34] Akila P, Prashant V, Suma MN, Prashant SN, Chaitra TR (2012). CD163 and its expanding functional repertoire. Clin Chim Acta..

[35] Hu H, Hang JJ, Han T, Zhuo M, Jiao F, Wang LW (2016). The M2 phenotype of tumor-associated macrophages in the stroma confers a poor prognosis in pancreatic cancer. Tumour Biol..

[36] Shigeoka M, Urakawa N, Nakamura T, Nishio M, Watajima T, Kuroda D (2013). Tumor associated macrophage expressing CD204 is associated with tumor aggressiveness of esophageal squamous cell carcinoma. Cancer Sci..

[37] Thanee M, Loilome W, Techasen A, Namwat N, Boonmars T, Pairojkul C (2015). Quantitative changes in tumor-associated M2 macrophages characterize cholangiocarcinoma and their association with metastasis. Asian Pac J Cancer Prev..

[38] Kamper P, Bendix K, Hamilton-Dutoit S, Honoré B, Nyengaard JR, d'Amore F (2011). Tumor-infiltrating macrophages correlate with adverse prognosis and Epstein-Barr virus status in classical Hodgkin's lymphoma. Haematologica..

[39] Kim KJ, Wen XY, Yang HK, Kim WH, Kang GH (2015). Prognostic implication of M2 macrophages are determined by the proportional balance of tumor associated macrophages and tumor infiltrating lymphocytes in microsatellite-unstable gastric carcinoma. PLoS One..

[40] Popa ER, Tervaert JW (2003). The relation between Staphylococcus aureus and Wegener's granulomatosis: current knowledge and future directions. Intern Med..

[41] Krysko O, Holtappels G, Zhang N, Kubica M, Deswarte K, Derycke L (2011). Alternatively activated macrophages and impaired phagocytosis of S. aureus in chronic rhinosinusitis. Allergy.

[42] Popa ER, Stegeman CA, Abdulahad WH, van der Meer B, Arends J, Manson WM (2007). Staphylococcal toxic-shock-syndrome-toxin-1 as a risk factor for disease relapse in Wegener's granulomatosis. Rheumatology (Oxford).

[43] Gadola SD, Gross WL (2012). Vasculitis in 2011: the renaissance of granulomatous inflammation in AAV. Nat Rev Rheumatol..

[44] Zhao Y, Odell E, Choong LM, Barone F, Fields P, Wilkins B (2012). Granulomatosis with polyangiitis involves sustained mucosal inflammation that is rich in B-cell survival factors and autoantigen. Rheumatology (Oxford).

