# Supplementary material for: M2 macrophage is the predominant phenotype in airways inflammatory lesions in patients with granulomatosis with polyangiitis
Source: Arthritis Res Ther. 2017 May 18;19:100. doi: 10.1186/s13075-017-1310-4 (PMC5437644; doi:10.1186/s13075-017-1310-4)
Supplement: Supplementary file 1 — Analysis of lymphocytes and macrophages in relation to disease parameters and nasal culture in GPA. Frequencies of CD68, CD86, CD163, CD3 and CD20 were compared between patients with GPA with localized and generalized disease and between patients with GPA who underwent airways biopsy at disease onset and during a disease relapse; no significant differences were observed. Evaluation of lymphocyte and macrophage scores in relation to positive and negative nose cultures for S. aureus yielded a significantly higher frequency of T cells in airway lesions from patients with GPA with a positive nasal culture for S. aureus compared with patients with GPA with a negative nasal culture for S. aureus. No significant differences were observed in B cell or macrophage frequencies in positivity of nasal culture for S. aureus in the airways of patients with GPA. (PDF 248 kb) [file 13075_2017_1310_MOESM1_ESM.pdf]

Table S1 – Analysis of lymphocytes and macrophages in relation to disease extension, disease stage and positivity of nasal culture in GPA.

Variables	Groups		
**Disease extension**	**Generalized (n=6)**	**Localized (n=29)**	***p***
CD3, %	5.9 (4.8-10.8)	7.9 (5.4-14.8)	0.336
CD20, %	2.7 (1.6-8.0)	4.3 (2.5-9.1)	0.358
CD68, %	9.8 (6.0-20.5)	8.9 (6.7-18.1)	0.896
CD86, %	13.1 (5.7-28.1)	19.6 (10.0-34.2)	0.314
CD163, %	33.4 (21.9-47.9)	32.4 (20.9-41.9)	0.965
**Disease stage**	**Disease onset (n=9)**	**Relapse (n=25)**	***p***
CD3, %	7.9 (5.9-10.8)	7.2 (5.1-14.8)	0.740
CD20, %	5.9 (2.9-11.6)	3.9 (1.9-8.5)	0.160
CD68, %	11.2 (6.8-20.3)	8.6 (5.8-15.6)	0.545
CD86, %	16.0 (6.6-26.9)	18.2 (11.6-39.3)	0.219
CD163, %	31.3 (23.5-39.7)	32.4 (20.2-41.4)	0.953
**Nasal culture for *S aureus***	**Positive (n=19)**	**Negative (n=15)**	***p***
CD3, %	10.5 (6.6-15.4)	5.9 (4.8-7.4)	0.014*
CD20, %	4.3 (2.5-7.6)	3.1 (1.9-9.7)	0.579
CD68, %	7.7 (5.2-17.1)	8.9 (7.7-23.4)	0.323
CD86, %	18.2 (7.4-38.0)	20.6 (12.5-29.5)	0.521
CD163, %	33.7 ± 15.0	37.4 ± 19.3	0.537

Data are presented as median and interquartile range. One patient with persisting disease was excluded from this analysis. Data are presented as median and interquartile range.
