# Supplementary material for: M2 macrophage is the predominant phenotype in airways inflammatory lesions in patients with granulomatosis with polyangiitis
Source: Arthritis Res Ther. 2017 May 18;19:100. doi: 10.1186/s13075-017-1310-4 (PMC5437644; doi:10.1186/s13075-017-1310-4)
Supplement: Supplementary file 2 — Evaluation of lymphocyte and macrophage scores in relation to therapy for GPA. Comparisons between patients with GPA who were not on therapy and patients with GPA who were on either prednisolone or immunosuppressive agents did not yield significant results for CD3+, CD20+, CD68+, CD86+ or CD163+ cells in the airways. Evaluation of lymphocyte and macrophage scores in relation to the use of daily co-trimoxazole in patients with GPA with positive nose cultures for Staphylococcus aureus. No significant differences in CD3+, CD20+, CD68+, CD86+ or CD163+ cells in the airways were found between patients with GPA who were or were not on daily co-trimoxazole. Evaluation of lymphocyte and macrophage scores in relation to the use of daily co-trimoxazole in patients with GPA with negative nose cultures for S. aureus; no significant differences were found in CD3+, CD20+, CD68+, CD86+ or CD163+ cells in the airways between patients with GPA who were or were not on daily co-trimoxazole. Evaluation of lymphocyte and macrophage scores in relation to the use of prednisolone in patients with GPA; no significant differences in CD3+, CD20+, CD68+, CD86+ or CD163+ cells in the airways were found between patients with GPA who were or were not on prednisolone. (PDF 153 kb) [file 13075_2017_1310_MOESM2_ESM.pdf]

**Table S2** - Evaluation of lymphocyte and macrophage scores in relation to therapy for GPA.

| **Variables** | **Groups** | | |
|---|---|---|---|
| **Presence or absence of therapy** | **Therapy (n=13)** | **No therapy (n=22)** | ***p*** |
| CD3, % | 7.4 (6.2-10.8) | 7.3 (4.9-15.7) | 0.785 |
| CD20, % | 5.7 (2.7-8.0) | 3.9 (1.8-9.9) | 0.290 |
| CD68, % | 8.3 (6.8-17.3) | 9.2 (6.0-19.5) | 0.946 |
| CD86, % | 16.0 (7.4-26.9) | 20.0 (10.4-38.6) | 0.339 |
| CD163, % | 30.7 ± 15.2 | 37.9 ± 17.2 | 0.224 |
| **Co-trimoxazol in patients with positive nose culture for *S aureus*** | **Co-trimoxazol (n=7)** | **No co-trimoxazol (n=12)** | ***p*** |
| CD3, % | 13.4 ± 11.9 | 11.2 ± 5.0 | 0.575 |
| CD20, % | 2.5 (1.9-4.3) | 6.2 (3.0-9.6) | 0.117 |
| CD68, % | 6.3 (5.1-13.3) | 13.4 (6.6-18.7) | 0.083 |
| CD86, % | 18.2 (8.3-23.9) | 18.7 (6.3-38.7) | 0.966 |
| CD163, % | 31.1 ± 12.8 | 35.2 ± 16.5 | 0.585 |
| **Co-trimoxazol in patients with negative nose culture for *S aureus*** | **Co-trimoxazol (n=7)** | **No co-trimoxazol (n=8)** | ***p*** |
| CD3, % | 5.1 (3.6-8.9) | 6.2 (4.9-7.2) | 0.778 |
| CD20, % | 7.7 ± 7.9 | 4.1 ± 3.5 | 0.271 |
| CD68, % | 9.6 (8.6-20.7) | 8.1 (7.3-28.1) | 0.778 |
| CD86, % | 23.4 (13.0-40.7) | 18.8 (11.2-24.4) | 0.535 |
| CD163, % | 34.4 ± 14.3 | 39.9 ± 23.5 | 0.601 |
| **Use of prednisolone** | **Prednisolone (n=10)** | **No prednisolone (n=25)** | ***p*** |
| CD3, % | 5.4 (4.3-9.8) | 8.0 (6.2-14.8) | 0.068 |
| CD20, % | 5.6 (1.8-10.9) | 4.0 (2.5-7.4) | 0.827 |
| CD68, % | 15.1 (6.7-26.4) | 8.3 (6.3-14.8) | 0.144 |
| CD86, % | 23.7 (12.4-41.3) | 18.2 (8.1-24.2) | 0.243 |
| CD163, % | 43.7 ± 20.2 | 31.8 ± 14.0 | 0.055 |

Data are presented as mean and standard deviation or as median and interquartile range.
