# Supplementary material for: M2 macrophage is the predominant phenotype in airways inflammatory lesions in patients with granulomatosis with polyangiitis
Source: Arthritis Res Ther. 2017 May 18;19:100. doi: 10.1186/s13075-017-1310-4 (PMC5437644; doi:10.1186/s13075-017-1310-4)
Supplement: Supplementary file 3 — Correlation between prednisolone daily dose and lymphocytes and macrophages in airway biopsies from patients with GPA. Significant correlation was found between daily dose of prednisolone in patients with GPA and all macrophage markers. (PDF 235 kb) [file 13075_2017_1310_MOESM3_ESM.pdf]

Table S3 – Correlations of lymphocytes and macrophages in airway biopsies with prednisolone daily dose in GPA patients.

**Variables**	**Daily prednisolone dose in mg**
CD3	Rho = 0.006; *p* = 0.986
CD20	Rho = -0.006; *p* = 0.986
CD68	Rho = 0.858; *p* = 0.001*
CD86	Rho = 0.753; *p* = 0.012*
CD163	Rho = 0.759; *p* = 0.011*

*Significant correlations.
