# Supplementary material for: M2 macrophage is the predominant phenotype in airways inflammatory lesions in patients with granulomatosis with polyangiitis
Source: Arthritis Res Ther. 2017 May 18;19:100. doi: 10.1186/s13075-017-1310-4 (PMC5437644; doi:10.1186/s13075-017-1310-4)
Supplement: Supplementary file 4 — Univariate linear regression to analyze associations between macrophage markers and therapy in patients with GPA. Significant associations were found between all macrophage markers and daily dose of prednisolone and the use of immunosuppressive agents. (PDF 316 kb) [file 13075_2017_1310_MOESM4_ESM.pdf]

Table S4 – Univariate linear regression to analyze associations between macrophage markers and therapy in GPA patients.

**Variables**	**β coefficient**	**R^2**	***p***
CD68 and prednisolone dose	1.16	0.739	0.001
CD86 and prednisolone dose	1.53	0.723	0.002
CD163 and prednisolone dose	1.48	0.545	0.015
CD68 and immunosuppressive agents	7.26	0.138	0.028
CD86 and immunosuppressive agents	11.49	0.153	0.020
CD163 and immunosuppressive agents	14.64	0.185	0.010
