# Supplementary material for: M2 macrophage is the predominant phenotype in airways inflammatory lesions in patients with granulomatosis with polyangiitis
Source: Arthritis Res Ther. 2017 May 18;19:100. doi: 10.1186/s13075-017-1310-4 (PMC5437644; doi:10.1186/s13075-017-1310-4)
Supplement: Supplementary file 5 — Multivariate linear regression models to analyze associations between CD3, CD20, CD68, CD86 and CD163 scores in airway biopsies and disease parameters/therapy in patients with GPA. No significant associations were observed between macrophages, B cell or T cell markers with age, gender, positive nasal culture for S. aureus, disease extension, use of co-trimoxazole, daily dose of prednisolone or use of immunosuppressive agents. (PDF 240 kb) [file 13075_2017_1310_MOESM5_ESM.pdf]

Table S5 - Multivariate linear regression models to analyze associations between CD3, CD20, CD68, CD86 and CD163 scores in airway biopsies and disease parameters/therapy in GPA patients.

**Associations between CD3 scores and disease parameters/therapy**		
**Variables**	**β coefficient**	***p***
Age	-0.53	0.292
Female gender	1.39	0.836
Positive nasal culture for *Staphylococcus aureus*	-4.52	0.492
Generalized disease	4.29	0.606
Use of co-trimoxazole	3.58	0.561
Daily dose of prednisone, mg	0.10	0.788
Use of immunosuppressive agents	18.85	0.201
**Associations between CD20 scores and disease parameters/therapy**		
**Variables**	**β coefficient**	***p***
Age	-1.37	0.156
Female gender	-12.30	0.328
Positive nasal culture for *Staphylococcus aureus*	-13.43	0.267
Generalized disease	20.46	0.217
Use of co-trimoxazole	18.59	0.158
Daily dose of prednisone, mg	1.03	0.201
Use of immunosuppressive agents	23.73	0.282
**Associations between CD68 scores and disease parameters/therapy**		
**Variables**	**β coefficient**	***p***
Age	0.28	0.552
Female gender	20.66	0.080
Positive nasal culture for *Staphylococcus aureus*	9.82	0.228
Generalized disease	-16.40	0.159
Use of co-trimoxazole	-10.94	0.184
Daily dose of prednisone, mg	0.59	0.238
Use of immunosuppressive agents	6.05	0.625
**Associations between CD86 scores and disease parameters/therapy**		
**Variables**	**β coefficient**	***p***
Age	-0.64	0.648
Female gender	-0.60	0.977
Positive nasal culture for *Staphylococcus aureus*	-14.03	0.504
Generalized disease	16.40	0.545
Use of co-trimoxazole	9.94	0.610
Daily dose of prednisone, mg	1.89	0.223
Use of immunosuppressive agents	14.95	0.687
**Associations between CD163 scores and disease parameters/therapy**		
**Variables**	**β coefficient**	***p***
Age	0.45	0.697
Female gender	36.48	0.148
Positive nasal culture for *Staphylococcus aureus*	9.65	0.576
Generalized disease	-20.96	0.385
Use of co-trimoxazole	-23.27	0.237
Daily dose of prednisone, mg	0.30	0.771
Use of immunosuppressive agents	19.53	0.544
